# Hypoxic stress suppresses lung tumor-secreted exosomal miR101 to activate macrophages and induce inflammation

**DOI:** 10.1038/s41419-021-04030-x

**Published:** 2021-08-06

**Authors:** Jie Li, Peng Xu, Di Wu, Minjie Guan, Xuanwen Weng, Yongzhen Lu, Yuwei Zeng, Rongchang Chen

**Affiliations:** Key Laboratory of Shenzhen Respiratory Disease, Shenzhen Institute of Respiratory Disease, Shenzhen People’s Hospital (The First Affiliated Hospital of Southern University of Science and Technology, The Second Clinical Medical College of Jinan University), Shenzhen, Guangdong China

**Keywords:** Small-cell lung cancer, Small-cell lung cancer

## Abstract

Hypoxia promotes inflammation in the tumor microenvironment. Although hypoxia-inducible factor 1α (HIF1α) is a master modulator of the response to hypoxia, the exact mechanisms through which HIF1α regulates the induction of inflammation remain largely unclear. Using The Cancer Genome Atlas Lung Squamous Cell Carcinoma (TCGA-LUSC) database, we divided patients with LUSC into two groups based on low or high HIF1α expression. After analyzing the differentially expressed genes in these two groups, we found that HIF1α was positively correlated with interleukin 1A (IL1A) and IL6 expression. Our in vitro study showed that hypoxic stress did not induce IL1A or IL6 expression in tumor cells or macrophages but dramatically enhanced their expression when co-cultured with tumor cells. We then investigated the effect of tumor-derived exosomes on macrophages. Our data suggested that the changes in miR101 in the tumor-derived exosomes played an important role in IL1A and IL6 expression in macrophages, although the hypoxic stress did not change the total amount of exosome secretion. The expression of miR101 in exosomes was suppressed by hypoxic stress, since depletion of HIF1α in tumor cells recovered the miR101 expression in both tumor cells and exosomes. In vitro, miRNA101 overexpression or uptake enriched exosomes by macrophages suppressed their reprogramming into a pro-inflammatory state by targeting CDK8. Injection of miR101 into xenografted tumors resulted in the suppression of tumor growth and macrophage tumor infiltration in vivo. Collectively, this study suggests that the HIF1α-dependent suppression of exosome miR101 from hypoxic tumor cells activates macrophages to induce inflammation in the tumor microenvironment.

## Introduction

A hypoxic tumor microenvironment, a prevalent characteristic of solid tumors, is related to aggressiveness and unfavorable prognosis [1]. This condition can trigger genes associated with modulation of cell proliferation and adhesion and extracellular matrix production. The underlying mechanism is related to the induction of the hypoxia-inducible factor (HIF) family of transcription factors, including HIF-1, -2, and -3 [2]. Accumulating evidence indicates that hypoxia can induce inflammation in the tumor microenvironment, which contributes to tumor progression [3]. Macrophages are major components of the immune infiltrate in solid tumors and can differentiate into tumor-associated macrophages (TAMs), which mainly exist in the hypoxic zone of the tumor [4]. Cytokines derived from tumors can convert TAMs into polarized M2 macrophages with more substantial anti-immunity effects, resulting in tumor development. Considering the pivotal role of hypoxia in the modulation of tumor development and immune inhibition, it is worthy of further study for the development of novel cancer treatments. Therefore, it is critical to investigate the interactions of macrophages and cancer cells in the hypoxic microenvironment, as it is valuable for studying cancer progression and treatment responsiveness.

Exosomes, first thought of as “garbage bins” for worthless cell materials, are now known to exhibit various functions, including interactions between cellular microenvironments, owing to their ability to carry several substances, including nucleic acids, lipids, proteins, and metabolites [5, 6]. Exosome-mediated continuous interference between stroma and cancer cells was previously thought to modulate hypoxia adaptation and re-establish the microenvironment in return [7]. Hypoxia changes the nucleic acid and proteomic profiles of exosomes considerably, making them a promising nonintrusive biomarker for tumor hypoxia status [6, 7]. Therefore, exosome analysis is a pathway to discovering new nucleotide and protein biomarkers for tumor diagnosis and prognosis. Exosomal miRNAs, a main element of exosomes, are extensively involved in cancer development and prognosis [8, 9]. For example, exosome miR155 and miR10b are the key modulators of tumor chemoresistance or pathology [10, 11]. In addition, exosomal miR-25-3p abundance is related to the development of some cancers by modulating interactions among cells, such as kidney cancer and liposarcoma [12, 13]. Therefore, dysregulation of miRNA in tumor-secreted exosomes assumes a critical role in tumor progression and inflammation in the tumor microenvironment.

Here, we studied the effect of hypoxia on lung cancer progression using The Cancer Genome Atlas (TCGA) database analysis. We found that high hypoxic stress in lung cancer is correlated with inflammatory cytokine secretion and expression in the tumor microenvironment, including IL1A and IL6. The majority of IL1A and IL6 expression originated from macrophages when co-cultured with lung tumor cells. Hypoxia stress reduced the secretion of exosomal miR101 from tumor cells, which led to the induction of IL1A and IL6 in macrophages, in turn promoting lung tumor cell growth. Our results elucidated the mechanism by which hypoxia-induced inflammation in lung cancer, and revealed that modulation of exosomal miR101 expression could inhibit tumor inflammation and progression induced by hypoxic stress.

## Results

### Hypoxic stress in lung cancer is correlated with increased IL1A and IL6 expression

**Fig. 1:**
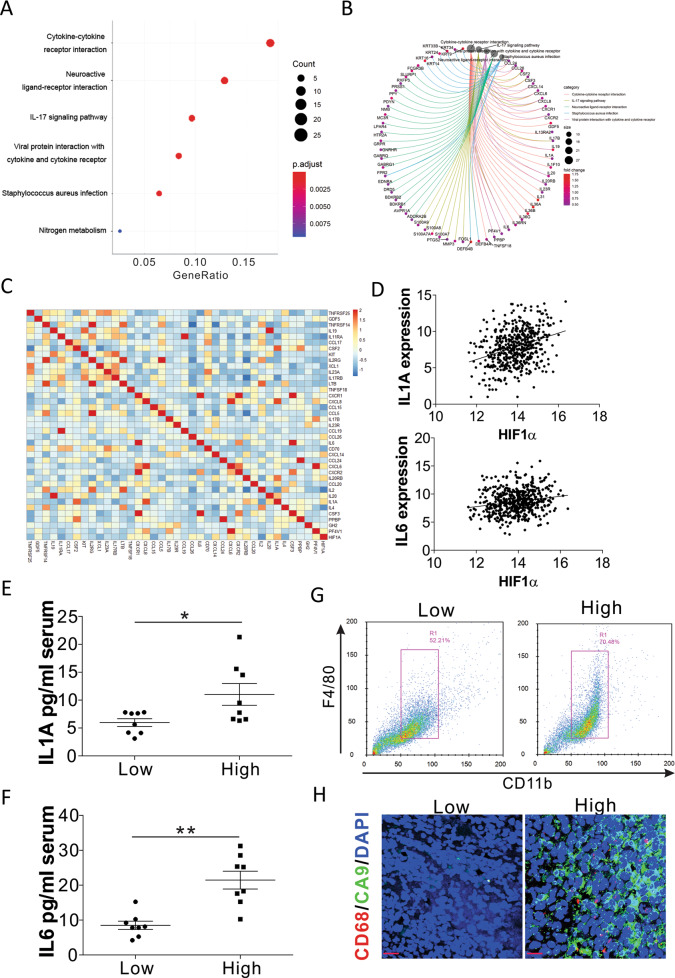
Hypoxic stress leads to inflammation in lung cancer. **A** Gene enrichment analysis on differentially expressed genes between LUSC tumors with low and high expression of HIF1α. **B** The groups of enriched genes in each pathway. **C** The heatmap of expression correlation of HIF1α with the genes in the cytokine-cytokine receptor interaction pathway. **D** The expression correlation of HIF1α and IL1A and IL6 from TCGA database (https://xena.ucsc.edu/welcome-to-ucsc-xena/). **E**, **F** The serum levels of IL1A (E) and IL6 (F) in lung cancer patients with low and high HIF1α expression in primary tumors (*n* = 8 in each group). **G** Flow cytometry analysis of macrophages in human lung tumors with low and high HIF1α expression. Representative images are shown. **H** The immunofluorescence staining of CD68 and CA9 in human lung tumors with low and high HIF1α expression. Scale bar, 20 μm. * *p* < 0.05; ** *p* < 0.01.

To study the effects of hypoxia on lung cancer development, we analyzed HIF1α expression in the normal and tumor tissues using the TCGA database. The higher expression of HIF1α in lung cancer tissues indicated that hypoxia might contribute to tumor progression in lung cancer (Fig. S1A). We then divided the patients into two groups based on their HIF1α expression level by using the median HIF1α expression as a cutoff value (Log(changes) = 0.456) and compared the differentially expressed genes. Overall, 1236 significantly downregulated and 430 significantly upregulated genes were identified in the HIF1α high-expression patients (Fig. S1B). The KEGG pathway enrichment analysis showed that the top function of upregulated genes was correlated with cytokine-cytokine receptor interactions (Fig. 1A), including several pro-inflammation-related cytokines, such as IL1A, IL6, IL19, IL17B, and IL20RB (Fig. 1B). These results indicated that higher hypoxic stress in tumor tissues might contribute to inflammation in the tumor microenvironment. We then analyzed the correlation of these cytokine-cytokine receptor interaction genes with the expression of HIF1α in the TCGA database using the R package. CCL26, IL20RB, IL20, CXCL8, CSF2, PF4V1, IL1A, and IL6 showed a significant positive correlation with the expression of HIF1α (Fig. 1C). As IL1A and IL6 are two important pro-inflammatory cytokines in human cancers [14], we speculated that the hypoxic stress in the lung tumor promotes the secretion of IL1A and IL6. The correlation of IL1A and IL6 with HIF1α was also validated by a linear regression plot (Fig. 1D). To further confirm the correlation between IL1A, IL6, and HIF1α, we analyzed the expression of these two genes in 16 patients with lung cancer. The secretion of IL1A and IL6 in the serum of patients with higher HIF1α mRNA expression was significantly higher than the patients with lower HIF1α mRNA levels in tumor samples (Fig. 1E, F, Fig. S1C). To investigate the correlation between inflammation and hypoxia in lung cancer, we analyzed the macrophage population in different groups of lung tumors. The tumors with higher HIF1α mRNA levels showed higher macrophage accumulation in the tumors (Fig. 1G). The immunostaining of CD68, the marker of macrophage, and carbonic anhydrase 9 (CA9), the marker of hypoxia, also indicated that higher macrophage accumulation in those tumors with higher hypoxia levels (Fig. 1H). Therefore, hypoxic stress might contribute to inflammation in the lung tumor microenvironment.

### Hypoxic stress in tumor cells stimulates the expression of IL1A and IL6 in macrophages

**Fig. 2:**
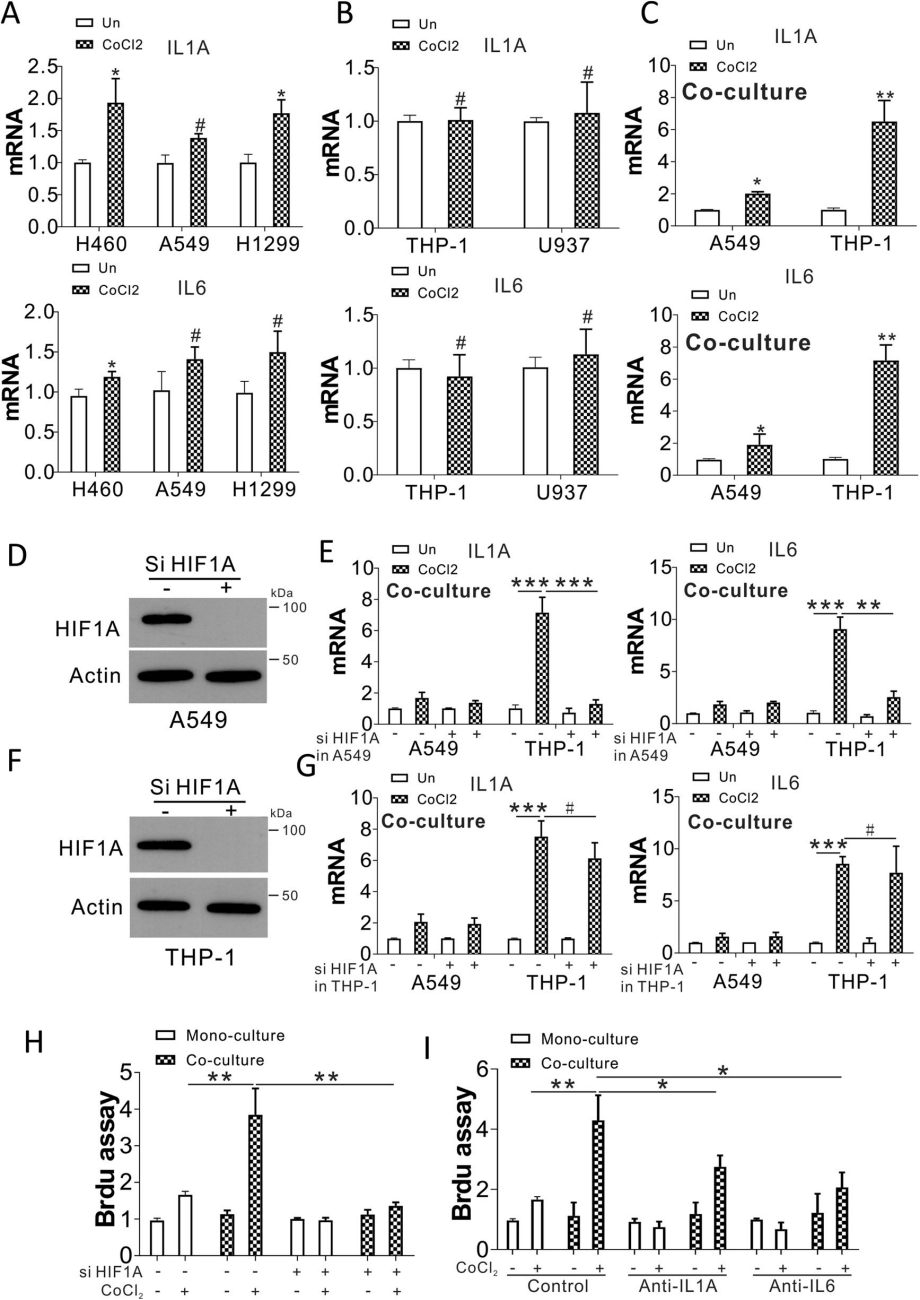
Hypoxic stress in tumor cells mediates IL1A and IL6 expression in macrophages. **A** IL1A and IL6 expression in H460, A549, and H1299 exposed to 100 µM CoCl_2_ for 24 h. **B** IL1A and IL6 expression in THP-1 and U937 cells exposed to 100 µM CoCl_2_ for 24 h. **C** IL1A and IL6 expression in A549 and THP-1 cells in the trans-well co-culture system with or without CoCl_2_ treatment in A549 cells. **D** HIF1α expression in A549 cells treated with HIF1α siRNA. **E** A549 cells transfected with control or HIF1α siRNA treated with CoCl_2_ for 1 d, and subsequently co-cultured with THP-1 cells for an additional day. IL1A and IL6 expression in A549 and THP-1 cells analyzed by RT-PCR. **F** HIF1α expression in THP-1 cells transfected with HIF1α siRNA. **G** A549 cells were treated with CoCl_2_ for 1 d and co-cultured with THP-1 cells with or without HIF1α siRNA transfection for an additional day. The expression of IL1A and IL6 in A549 and THP-1 cells was analyzed by RT-PCR. **H** The A549 cells transfected with control or HIF1α siRNA were treated with CoCl_2_ for 1 d, and co-cultured with THP-1 cells for an additional day. A549 proliferation was studied with a BrdU assay. **I** A549 cells were exposed to CoCl_2_ for 1 d, and co-cultured with THP-1 cells with or without anti-IL1A (MABp1, 10 ng/ml) or anti-IL6 (Sirukumab, 20 ng/ml) treatment for a further 24 h. A549 proliferation was studied using a BrdU assay. ^#^ *p* > 0.05; * *p* < 0.05; ** *p* < 0.01; *** *p* < 0.001.

To explore the mechanism by which hypoxia elicits inflammation in lung tumors, we studied IL1A and IL6 expression in tumor cells and macrophages in response to hypoxic stress. By treating the lung cancer cells, A549, H460, and H1299, with CoCl_2_, the mRNA levels of IL1A and IL6 were mildly induced (Fig. 2A). Treatment with CoCl_2_ did not change the expression of IL1A and IL6 in THP-1 and U937 cells (Fig. 2B). We then tested whether hypoxia-induced IL1A and IL6 expression in tumor cells and macrophages when co-cultured together. The expression of IL1A and IL6 was dramatically upregulated in THP-1 cells when co-cultured with CoCl_2_-treated A549 cells (Fig. 2C). To rule out the side effects of CoCl_2_, we also confirmed the effect of hypoxia in tumor cells and macrophages by using normoxic and hypoxic culture conditions, and similar results were found (Fig. S2A–C). Furthermore, the induction of IL1A and IL6 was found in THP-1 cells when co-cultured with CoCl_2_-treated H460 and H1299 cells (Fig. S2D, E). To further confirm the effect of hypoxia on the cytokine expression of macrophages co-cultured with tumor cells, we knocked down the hypoxia modulator, HIF1α, in A549 cells and analyzed the expression of IL1A and IL6. In the A549 and THP-1 co-culture system, HIF1α siRNA transfection in A549 cells mildly suppressed the induction of IL1A and IL6 in A549 cells under hypoxia stress, but significantly inhibited the induction of IL1A and IL6 in THP-1 cells (Fig. 2D, E). However, depletion of HIF1α in THP-1 did not affect the induction of IL1A and IL6 by CoCl_2_ in both A549 and THP-1 cells in the co-culture system (Fig. 2F, G). In terms of A549 cell growth, A549 cell proliferation was maximally induced under hypoxic stress when co-cultured with THP-1 cells (Fig. 2H). This scenario was abolished by transfection of HIF1α siRNA in A549 cells (Fig. 2H), and partially blocked by treating with anti-IL1A (MABp1, 10 ng/ml) or anti-IL6 (Sirukumab, 20 ng/ml) (Fig. 2I). These results collectively suggest that hypoxia in tumor cells initiates the expression of IL1A and IL6 in macrophages, which leads to inflammation in the tumor microenvironment.

### Exosomes secreted by tumor cells stimulate IL1A and IL6 expression in macrophages

**Fig. 3:**
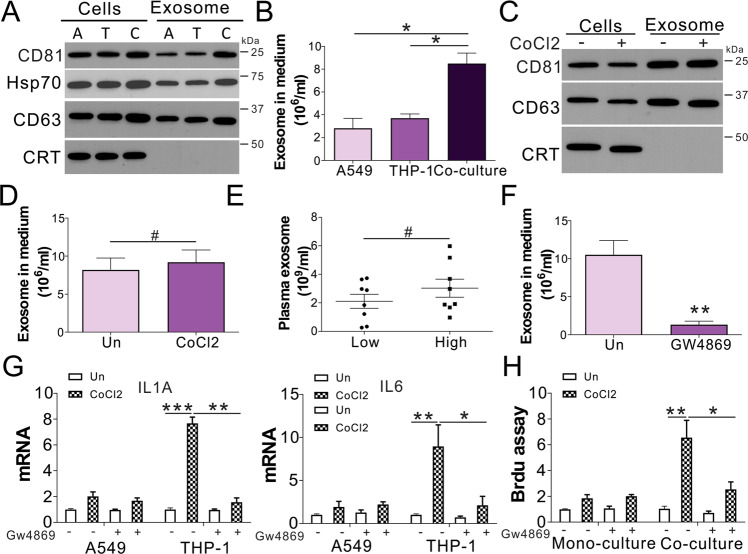
Exosomes secreted by lung cancer cells trigger macrophages and promote inflammation. **A** The exosomal positive markers CD81, CD63, and Hsp90 were observed in cells and media of A549, THP-1 mono-, and co-culture. Calreticulin expression served as a negative marker. A: A548 cells; T: THP-1 cells; C: co-culture. **B** Exosome levels in the cell culture medium of A549, THP-1 mono-, and co-culture. **C** A549 cells were treated with CoCl_2_ for 1 d, and co-cultured with THP-1 cells for another day. CD63, CD81, and CRT expression in co-cultured cells and media were studied through western blotting. **D** Exosome levels in the cell culture medium in (**C**). **E** Exosome levels in the plasma of patients with lung cancer with low and high HIF1α expression (*n* = 8 in each group). **F** The exosome level in the medium of A549 co-cultured with THP-1 exposed to 10 µM GW4869. **G** A549 co-cultured with THP-1 exposed to 10 µM GW4869. IL1A and IL6 expression was studied through RT-PCR. **H** A549 cells were treated with CoCl_2_ for 1 d, and co-cultured with THP-1 cells with or without 10 µM GW4869 for a further 24 h. The growth of A549 was analyzed by BrdU assay. ^#^ *p* > 0.05; * *p* < 0.05; ** *p* < 0.01; *** *p* < 0.001.

Exosomes, considered pivotal regulators of cell-to-cell communication, abnormally increase in several cancers [15, 16]. We first examined whether lung tumor cells and macrophages secrete exosomes. Several exosome markers, including heat shock protein 90 (Hsp90), CD81, and CD63, were detected in exosomes isolated from A549 and THP-1 cells (Fig. 3A). Calreticulin (CRT), an intracellular contaminant, was negative (Fig. 3A). A549 and THP-1 co-culture enhanced exosome release (Fig. 3A). The enhanced exosome secretion in the co-culture system was also confirmed by Nanoparticle Tracking Analysis (NTA) (Fig. 3B). Next, we tested whether hypoxia in tumor cells changes exosome secretion when co-cultured with macrophages. The hypoxic stress in A549 cells did not alter the secretion of exosomes when co-cultured with THP-1 cells (Fig. 3C, D). The plasma exosome number in the serum of patients with lung cancer did not show any difference between groups with low and high expression of HIF1α (Fig. 3E). However, exosome release suppression by GW4869 inhibited IL1A and IL6 expression in macrophages (Fig. 3F, G). It also repressed A549 cell growth when co-cultured with THP-1 cells (Fig. 3H). Therefore, these findings revealed that exosome release contributes to hypoxia-induced macrophage inflammation.

### Reduction of miR101 in exosome mediates the interaction of tumor cells and macrophages

**Fig. 4:**
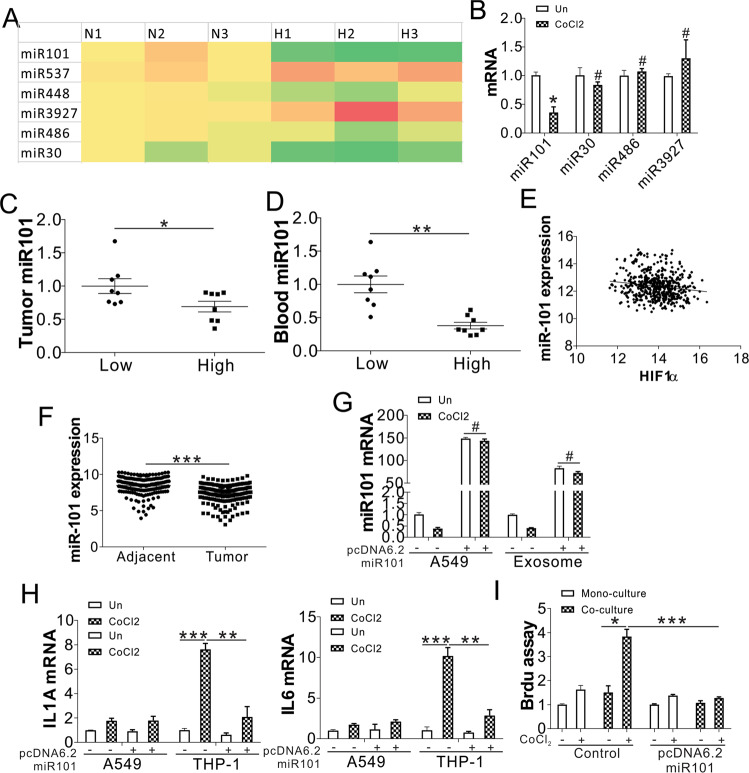
Hypoxic stress suppresses the exosomal miR101 to mediate the expression of IL1A and IL6 in macrophages. **A** The expression of miRNAs in A549 cells under normoxia and hypoxia. **B** The expression of miR101, miR30, and miR486, miR3927 in the exosomes of A549 cells treated with CoCl_2_. **C** The miR101 expression in human lung tumors with low and high HIF1α expression. **D** The miR101 expression in the blood of patients with lung cancer with low and high HIF1α expression in tumors. **E** The correlation between miR101 and HIF1α expression in the LUSC database. **F** The miR101 expression in lung adjacent and tumor samples from the GEO database (GSE15008). **G** A549 cells transfected with pcdna6.2-gw/emgfp miR101 were treated with CoCl_2_ for 1 d. The mRNA level of miR101 in the A549 cells and exosome was analyzed. **H**, **I** A549 cells transfected with pcdna6.2-gw/emgfp miR101 were treated with CoCl_2_ for 1 d, and co-cultured with THP-1 cells. (**H**) The expression of IL1A and IL6 in A549 and THP-1 cells was analyzed by RT-PCR. **I** The growth of A549 was analyzed by BrdU assay. ^#^ *p* > 0.05; * *p* < 0.05; ** *p* < 0.01; *** *p* < 0.001.

As hypoxia did not change the secretion of the exosomes, we next examined whether there were changes in the contents of exosomes secreted by tumor cells. Typically, miRNAs are considered to exert effects in the cells where they are produced. Moreover, miRNAs have been recently detected in exosomes [11, 12] and have emerged as new modulators of cell functions. To investigate the role of miRNAs in lung cancer development, we compared the regulation of miRNAs in different stages (Stage I, T1; Stage II, T2; Stage III, T3; Stage IV, T4) of lung cancer with normal lung tissues using TCGA-LUSC database. In total, 164 miRNAs showed significant upregulation or downregulation in different stages of LUSC (Fig. S3A, B). Among these 164 miRNAs, 6 top changed miRNAs were chosen in our further study, including miR30, miR101, miR486, miR573, miR3927, and miR448 (Fig. S3B). We found that only miR101 was significantly downregulated in A549 cells under hypoxic stress (Fig. 4A). Consistently, miR101 was also downregulated in the exosome of A549 cells upon CoCl_2_ treatment (Fig. 4B). Moreover, miR101 expression was lower in the lung tumors and blood from the patients with lung cancer with higher HIF1α expression (Fig. 4C, D). TCGA data showed that miR101 expression was inversely related to HIF1α expression (Fig. 4E). Depletion of HIF1α in A549 cells rescued the expression of miR101 in tumor cells and exosomes (Fig. S3C, D). The expression level of miR101 was also lower in the lung tumor samples, based on data analysis from the Gene Expression Omnibus (GEO) database (GSE15008) (Fig. 4F), further indicating the tumor-suppressive function of miR101. To investigate whether the dysregulation of miR101 in tumor cells affects the regulation of IL1A and IL6 in macrophages, we overexpressed miR101 in A549 cells by transfection of pcdna6.2-gw/emgfp miR101 plasmid. The transfection of miR101 plasmid dramatically increased the miR101 level in the A549 cells and the exosome (Fig. 4G), which was not affected by CoCl_2_ treatment. The transfection of miR101 plasmid in A549 cells suppressed the induction of IL1A and IL6 in THP-1 co-cultured with CoCl_2_-treated A549 cells (Fig. 4H). However, the miR101 plasmid did not have any effect on the expression of IL1A and IL6 in A549 cells upon CoCl_2_ treatment (Fig. 4H). The transfection of miR101 plasmid also compromised A549 cell growth with or without co-culture with THP-1 cells (Fig. 4H), further confirming its tumor-suppressive role. The administration of miR101 mimic in A549 and THP-1 co-culture system also had similar effects on IL1A and IL6 expression in THP-1 cells (Fig. S3E), and A549 cell proliferation (Fig. S3F). Collectively, our data indicate that hypoxic stress affects exosome miR101 levels, leading to the induction of IL1A and IL6 in macrophages.

### Exosomal miR101 targets CDK8 and SUB1 in mediating IL1A and IL6 expression

**Fig. 5:**
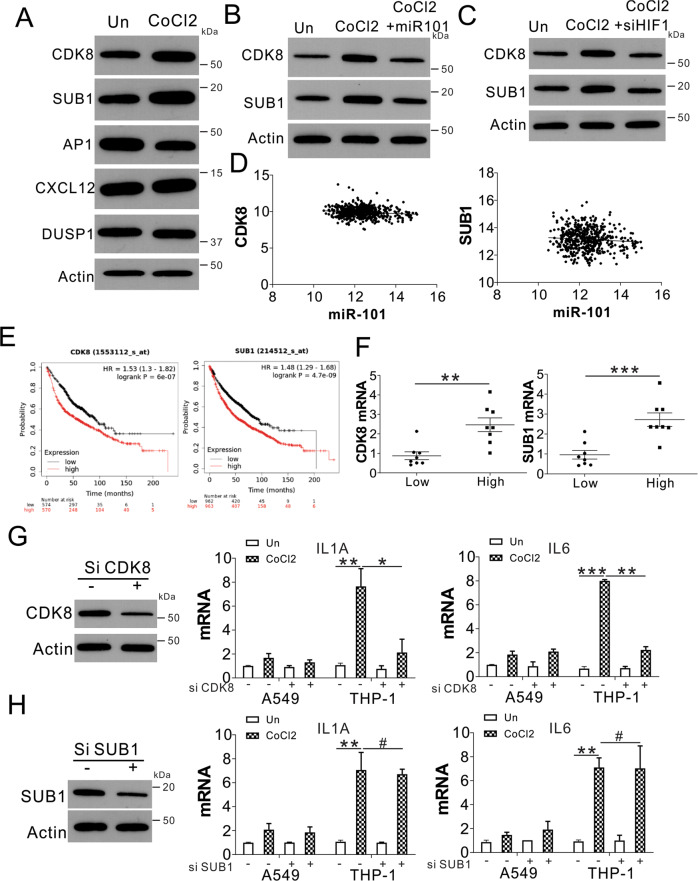
The exosomal miR101 targets CDK8 in mediating IL1A and IL6 expression. **A** The expression of indicated proteins in THP-1 cells co-cultured with control- or CoCl_2_-treated A549 cells. **B** THP-1 cells co-cultured with control- or CoCl_2_-treated A549 cells were transfected with or without miR101 mimic. The expression of CDK8 and SUB1 in THP-1 was analyzed by western blot. **C** THP-1 cells co-cultured with control- or CoCl_2_-treated A549 cells were transfected with or without HIF1α siRNA. The expression of CDK8 and SUB1 in THP-1 was analyzed by western blot. **D** The correlation of CDK8 and SUB1 expression with miR101 expression in the LUSC database. **E** The survival of patients with lung cancer with different expression levels of CDK8 and SUB1 from Kaplan-Meier plotter (https://kmplot.com/analysis/). **F** The expression of SUB1 and CDK8 in lung tumors with different levels of HIF1α expression. **G** The THP-1 cells were transfected with CDK8 siRNA and co-cultured with CoCl_2_-treated A549 cells. Left, the expression of CDK8 in THP-1 cells; right, the mRNA levels of IL1A and IL6 in THP-1 cells. **H** The THP-1 cells were transfected with SUB1 siRNA and co-cultured with CoCl_2_-treated A549 cells. Left, the expression of SUB in THP-1 cells; right, the mRNA levels of IL1A and IL6 in THP-1 cells. ^#^ *p* > 0.05; * *p* < 0.05; ** *p* < 0.01; *** *p* < 0.001.

Next, we investigated the mechanism by which miR101 mediates the expression of IL1A and IL6 in macrophages. We first analyzed the expression of several miR101 targets in THP-1 cells co-cultured with A549 with or without CoCl_2_ treatment, including CDK8, SUB1, AP1, CXCL12, DUSP1 [17]. We found that only CDK8 and SUB1 were upregulated in THP-1 cells co-cultured with A549 with CoCl_2_ treatment (Fig. 5A). A supplement of miR101 mimic or transfection of HIF1α in A549 cells compromised the upregulation of CDK8 and SUB1 in THP-1 cells co-cultured with CoCl_2_ treated A549 cells (Fig. 5B, C). The transfection of miR101 in THP-1 cells also suppressed the CDK8 and SUB1 expression in the mRNA level (Fig. S4A). TCGA database showed that CDK8 and SUB1 expression was inversely correlated with miR101 expression (Fig. 5D), but positively correlated with the expression of HIF1α (Fig. S4B, C). The high expression of CDK8 and SUB1 was correlated with the poor survival of patients with lung cancer (Fig. 5E). The expression of CDK8 and SUB1 was also higher in lung cancer patients with high HIF1α expression (Fig. 5F), further suggesting the upregulation of CDK8 and SUB1 by hypoxic stress. To further investigate the role of CDK8 and SUB1 in IL1A and IL6 expression in macrophages, we transfected the THP-1 cells with CDK8 or SUB1 siRNA. Knockdown of CDK8 by siRNA suppressed the induction of IL1A and IL6 in THP-1 cells co-cultured with A549 cells with CoCl_2_ treatment (Fig. 5G). However, it did not affect the mild induction of IL1A and IL6 in A549 cells (Fig. 5G). In contrast, the silence of SUB1 did not achieve as similar an effect as CDK8 (Fig. 5H). Finally, the CDK8 expression was also higher in the HIF1α high-expression lung tumor tissues (Fig. S4D). Therefore, our data suggest that CDK8 is the target of exosomal miR101 in mediating IL1A and IL6 expression in macrophages.

### Enhanced miR101 expression suppresses lung tumor growth and inflammation in vivo

**Fig. 6:**
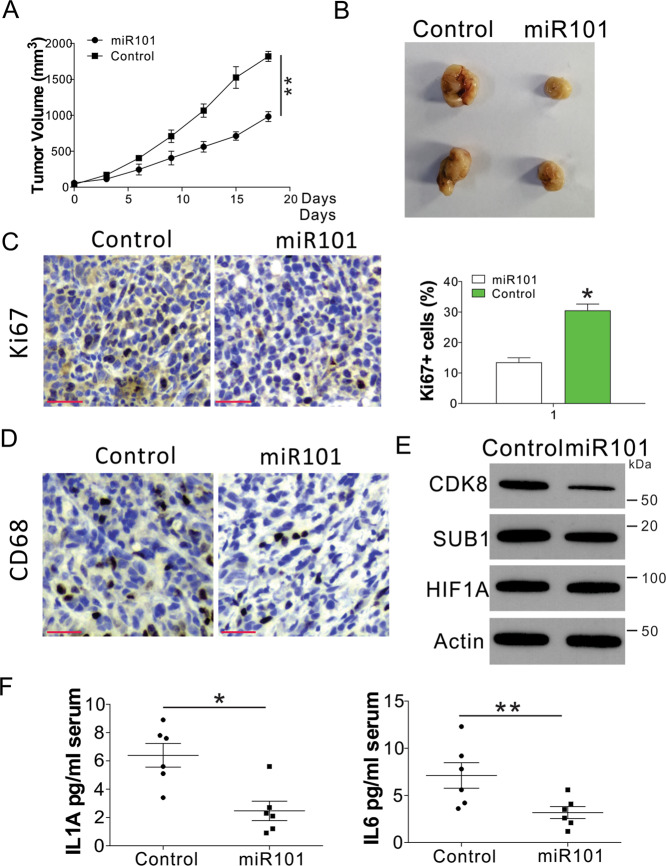
Administration of miR101 suppresses tumor growth and inflammation. **A** The tumor growth of LLC xenografted tumors with or without intratumor injection of the miR101 mimic. *n* = 6 for each group of mice. **B** Representative tumors. **C** Ki-67 staining of tumors. Scale bar, 50 μm. **D** CD68 staining of tumors. Scale bar, 50 μm. **E** The expression of indicated proteins in each group of tumors. **F** The serum levels of IL1A and IL6 in the different groups of mice. * *p* < 0.05; ** *p* < 0.01.

To study the effect of miR101 expression on lung tumor growth and inflammation, we xenografted the B56C/J mice with LLC mouse lung tumor cells. After tumor initiation, we treated the mice with miR101 mimic by intratumor injection. We found that the administration of miR101 significantly slowed tumor growth in vivo (Fig. 6A, B). Ki-67 staining indicated that the proliferation of the miR101-treated tumors was lower (Fig. 6C). CD68 staining suggested that miR101 suppressed macrophage accumulation in tumors (Fig. 6D). Furthermore, miR101 treatment suppressed the expression of CDK8 and Ki-67 but did not affect the expression of HIF1α (Fig. 6E). The administration of miR101 also suppressed serum IL1A and IL6 levels in mice (Fig. 6F). Collectively, our data indicate that the miR101 mimic could suppress lung tumor growth and inflammation in vivo.

## Discussion

Lung cancer seriously endangers public health with a reasonably unfavorable prognosis, and there is a lack of efficient treatment. Most solid tumors, including lung cancer, have regions permanently or transiently subjected to hypoxia [18]. Inflammation is another aspect of the tumor microenvironment that exhibits an important role in neoplastic development [3]. The hypoxic activation of tumor-promoting inflammatory responses is generally accepted [19]. Therefore, future studies should investigate the interference of hypoxic stress in tumor cells and inflammation in tumor microenvironments. This study proposes a new mechanism, whereby hypoxia elicits inflammation in the tumor microenvironment by inhibiting exosomal miR101, which stimulates IL1A and IL6 expression in macrophages, leading to inflammation.

Interactions among cells are important for immune cells coordinating inflammatory responses [20]. Cytokines, cell surface receptors, and chemokines are well-known regulators. Aside from several typical signaling molecules, increasing evidence indicates that immune cells are able to signal by releasing exosomes, which carry many different molecules that can be taken up by recipient cells [21]. Exosomes are notably complex vesicles that contain different types of membranes, soluble proteins, and RNAs, including miRNAs [21]. Exosomal transferred miRNAs are being recognized as new modulators of cell functions. Evidence suggests that transferred miRNAs inhibit target mRNAs in recipient cells [22, 23]. The miRNA transfer can also lead to physiological alterations in recipient cells [24, 25]. Recently, hypoxic pancreatic cancer cells were reported to be capable of promoting macrophages to the M2 phenotype by delivering miR-301a-3p that can initiate the PTEN/PI3Kγ pathway [26]. In addition, Chen et al. reported that hypoxia eliciting miR940 expression in exosomes stems from ovarian epithelial carcinoma, thereby promoting M2 phenotype polarization [27]. These findings indicate that hypoxia pressure on tumor cells alters the exosome miRNA profiles, which is capable of modulating macrophage differentiation and function in tumor cells. Our results show that hypoxic stress in tumor cells leads to the suppression of exosomal miR101, but does not change the secretion of total exosomes. Consistently, we have shown that downregulation of circulating exosomal miR101 is tightly related to the hypoxic status in patients with lung cancer. The suppression of exosomal miR101 stimulates the expression and secretion of IL1A and IL6 in macrophages and leads to inflammation in the tumor microenvironment. These findings further reveal that hypoxic exosomes are critical components of the tumor microenvironment and function as messengers that regulate cross-talk between cells.

miRNAs modulate the translation of specific protein-coding genes. miRNA expression is strictly controlled, and its expression dysregulation is associated with cancer [28]. Accumulating data reveal that miRNAs act as pivotal regulators linking cancer and inflammation [29]. The present study revealed that miR101, an anti-tumor miRNA, is reduced by hypoxic stress and acts as a pivotal regulator linking hypoxia and inflammation in lung cancer. As a tumor inhibitor miRNA, miR101 levels are frequently decreased in cancer, and they can target multiple oncogenes [30, 31]. It has also been reported that suppression of miR101 by IL1β to induce hypoxia is crucial for inflammation-promoted lung tumorigenesis [30]. However, it remains unclear whether suppression of miR101 by hypoxia can induce tumor microenvironment inflammation. Here, we demonstrated that hypoxia suppresses miR101 levels in lung cancer cells and derived exosomes, which was modulated by HIF1α. The suppression of exosomal miR101 enhanced CDK8 expression, which promotes IL1A and IL6 expression in macrophages. The supplementation of miR101 mimic impaired IL1A and IL6 expression in macrophages, which suppress lung tumor cell growth in vitro and in vivo. These findings indicate that lung cancer cell-derived exosomal miR101 assumes an important role in tumor microenvironment interactions and growth.

Tumor inhibitor miRNA deprivation is a confirmed mechanism in cancer development. Previous studies have shown that miR101 is capable of targeting other important genes, such as *EZH2*, *COX2*, *POMP*, *CERS6*, *STMN1*, *MCL-1*, and *ROCK2* [17]. The function of these targets is mostly correlated with tumor cell self-proliferation and migration. In this study, we revealed that CDK8, as the target of miR101, is involved in the expression of IL1A and IL6 by macrophages. An increasing number of studies have provided evidence for CDK8 as a coactivator in some transcription programs. For instance, CDK8 assumes a critical regulatory role in biological processes at the transcriptional level in the Wnt/β-catenin pathway and may be a proto-oncogene in human colon cancer [32, 33]. A recent study has shown that CDK8 together with transcription factors nuclear factor κ‐light‐chain enhancer of activated B cells (NF‐κB) and CCAAT/enhancer‐binding protein β (C/EBPβ) activate the transcription of inflammatory cytokines [34]. Usually, the NF-κB pathway modulates the release of pro-inflammatory cytokines, including IL1A and IL6, which are two critical inflammatory cytokines in macrophages [35, 36] and associated with unfavorable outcomes. Our data demonstrate that CDK8 mediates the expression of IL1A and IL6 in macrophages when co-cultured with hypoxia-treated lung tumor cells. Future studies should be directed toward a better understanding of the precise molecular mechanisms by which CDK8 mediates cytokine expression in macrophages in response to hypoxic stress.

This study first indicated that the tumor inhibitor miR101, which is suppressed in exosomes released by lung cancer cells under hypoxic stress, can trigger macrophages and promote inflammation. However, the present study had the limitation of being a relatively small retrospective cohort study from a single institute. Therefore, further study with a large cohort or prospective clinical trial with more extended follow-up periods is needed to validate these results. Furthermore, tumor suppressor miRNAs with more powerful anticancer effects could be identified by examining tumor suppressor miRNAs depleted in the plasma of patients with various cancer types using strategies such as microarray analysis, next-generation sequencing, or digital PCR-based approaches.

## Materials and methods

### Ethics

This study has been approved by the Ethics Committee of Shenzhen People’s Hospital (The First Affiliated Hospital of Southern University of Science and Technology, The Second Clinical Medical College of Jinan University). All study participants provided written informed consent before participating in the study.

### Data analysis

Lung squamous cell carcinoma (LUSC) RNA-seq and miRNA expression levels were acquired through TCGA (https://tcga-data.nci.nih.gov/docs/publications/tcga/). After the first filtering (eliminating samples with uncertain tumor stage), 513 samples were selected for the study. Clinical data, involving results and staging information, were also acquired. In total, 475 samples were LUSC samples (106 with T1 stage, 279 with T2 stage, 69 with T3 stage, and 21 with T4 stage) and 38 were healthy samples. Gene raw read counts were utilized to analyze different expressions through DESeq2 (v.1.18.1) [37], an R package that takes advantage of a model on the basis of the negative binomial distribution and is extensively applied to the differential analysis of RNA-seq data. The additional filter strategy was conducted to exclude the low-quality differentially expressed genes (DEGs). For mRNA and miRNA, only DEGs that displayed reads per kilobase million or fragments per kilobase million larger than the threshold of one for at least 10% of samples were retained. Subsequently, the normal sample with different tumor stages was compared to determine common DEGs for further analysis. To investigate the effects of hypoxia on lung cancer progression, we divided the patients into two groups based on hypoxia HIF1α expression levels using the median HIF1α expression as a cutoff value (Log(changes) = 0.456), and compared the differentially expressed genes. The Kyoto Encyclopedia of Genes and Genomes (KEGG) database was used to identify the DEGs in biological pathways (c2.cp.kegg.v5.1.symbols.gmt) [38].

### Cell lines and human samples

Human non-small-cell lung carcinoma (NSCLC) cell lines H1299, A549, and H460, the human monocytes, THP-1 and U937 cells, and the mouse lung cancer cell line Lewis lung carcinoma (LLC) were purchased from the American Type Culture Collection (ATCC) (2017. 2) and cultured based on their guidance. These lines were identified by cell morphology and short tandem repeat analysis at Ji-Ying Inc. (Shanghai, China; June 2017).

For cell treatment, IL1b (10 ng/mL, PrimeGene), aspirin (1.0 mM, in dimethylsulfoxide; Sangon Biotech) and CoCl_2_ (400 mM, Sigma) were used.

NSCLC samples (*n* = 16) were harvested from the Shenzhen People’s Hospital (Shenzhen, China) during surgery. Samples were instantly snap-frozen and preserved at −80 °C. Sample collection was approved by the relevant hospital authorities. The Institutional Review Board approved tissue procurement and analysis.

### Immunohistochemistry and immunofluorescence staining

Immunohistochemistry was performed to assess macrophage infiltration in tumors using rabbit polyclonal antibody against Ki67, CD68, CDK8 (Abcam, Cambridge, UK). Immunohistochemistry was conducted through an automated protocol for the DISCOVERY XT automated slide staining system via Ultramap anti-rabbit horseradish peroxidase (HRP) and was detected through ChromoMap DAB (Ventana Medical Systems Inc., Tucson, AZ, USA). Hematoxylin II (Ventana-Roche, Tucson, AZ, USA) served as the counterstain. For the immunofluorescence staining, the rabbit antibody against CD68 and mouse antibody against CA9 (carbonic anhydrase 9, hypoxia marker, Abcam) were used. After stained with primary antibodies, the slides of lung tissues were stained with mouse and rabbit secondary antibodies conjugated with Alexa 488 or Alexa 594 (Thermofisher, Waltham, MA, USA) to visualize the signal.

### Immunoblot analyses

For immunoblot analysis, 10 μg protein was subjected to sodium dodecyl sulfate-polyacrylamide gel electrophoresis and transferred to an immobilon-P polyvinylidene difluoride membrane (EMD Millipore, Billerica, MA, USA). The membrane was incubated in blocking buffer for 60 min (Tris-buffered saline (TBS)-T, 5% fat-free dry milk), followed by incubation with the primary antibody at 4 °C overnight. After rinsing with TBS-T, the blot was incubated with HRP-conjugated secondary antibody and signals were obtained through a chemiluminescence western blotting substrate as per relevant guidelines (EMD Millipore). The antibodies used in the study were HIF1α, β-actin, hsp70 (Cell Signaling, Danvers, MA, USA), CD63, CRT, CDK8 (Abcam), SUB1, AP1, CXCL12, DUSP1, and Ki67 (Santa Cruz, Dallas, TX, USA). All antibodies were used at optimized dilutions.

### Brdu assay and cell co-culture system

The proliferation of lung cancer cell was analyzed by Brdu assay (Thermofisher) as described by manufacturer. The co-culture of lung cancer cells and macrophages was conducted using Corning trans-well inserts (Sigma-Aldrich, St. Louis, MO, USA) as previously described [39].

### Exosome isolation and procedures

For exosome isolation, the medium from one million A549, THP-1 cells mono-cultured or co-cultured for 24 h was used. The exosomes were isolated from conditioned medium through differential centrifugation. The initial spins were composed of a spin for 10 min at 1000*g*, a spin for 10 min at 2000*g*, and a spin for 0.5 h at 10,000*g*. The supernatants were retained and subsequently spun for 70 min at 100,000*g* and the pellet was added to 1 × phosphate-buffered saline (PBS) to dilute residual soluble factors, and then centrifuged for 70 min at 100,000*g*. The exosomes existed in the final pellet, which were added to culture media. A Thermo Scientific Sorvall Lynx 6000 with a T26-8 × 50 rotor or a Beckman ultracentrifuge with a TI75 fixed angle rotor was used. The exosome concentration was determined using nanoparticle tracking analysis (NTA). Briefly, the cell culture medium or the plasma samples were diluted 1:10 and visualized on the NanoSight NS300 nanoparticles detector instrument (Malvern, Westborough, MA, USA).

GW4869 (Sigma-Aldrich, St. Louis, MO, USA) is a neutral sphingomyelinase 2 inhibitor that has previously been utilized to prevent exosome release.

### RNA extraction and qPCR

RNA was isolated through Qiagen’s miRNeasy kit, as per the manufacturer’s instructions. Mature miRNA cDNA was formed through RT miRNA PCR kit using RNA (10 ng) from each sample (Exiqon). qPCR was conducted using SYBR green MasterMix with locked nucleic acid (LNA) primers for miR101 (Exiqon). The expression was normalized via U6. cDNA was formed with qScript with RNA (30 ng) from each sample (Quanta). qPCR was conducted through a pPCR MasterMix. The following were the primer sequences: IL1A, CGCCAATGACTCAGAGGAAGA and AGGGCGTCATTCAGGATGAA; IL6, CCACTCACCTCTTCAGAACG and CATCTTTGGAAGGTTCAGGTTG, GAPDH, CATGTTCCAATATGATTCCAC and CCTGGAAGATGGTGATG, miR101, TGGGCTACAGTACTGTGATA and TGCGTGTCGTGGAGTC, HIF1α, GTACCCTAACTAGCCGAGGAAGAA and GTGAATGTGGCCTGTGCAGT, CDK8, AAGTTGGCCGAGGCACTTAT and ATGCCGACATAGAGATCCCA, SUB1, TTCGAGAGCCCTGTCATCTT and TTGCCTTTAAAATCGCGAAC.

### RNA interference, plasmid transfection, and miRNA transfection

The siRNA duplexes used for suppressing SUB1 expression were provided by Qiagen, and CDK8, HIF1α SiGenome SMARTpool was provided by GE Dharmacon (Lafayette, CO, USA). miR101 mimics and control RNAs were provided by Ribobio (Guangzhou, China). The pcdna6.2-gw/emgfp miR101 plasmid was constructed by Ribobio (Guangzhou, China). Transfections were conducted using Lipofectamine RNAiMAX or Oligofectamine (Life Technologies). For RNA interference or miRNA transfection, lung cancer cells (1 × 10^5^ cells/well) were plated onto a six-well plate, and 0.5 d later, the cells were exposed to miRNAs or siRNA duplexes. The same transfection was conducted again after 1 d. The cells were collected for isolating RNA or analyzing immunoblots 3 d after the first transfection.

### Tumor xenograft model

All operations were performed according to Animal Care and Use Guidelines and have been approved by the Animal Research Committee of Shenzhen People’s Hospital (The First Affiliated Hospital of Southern University of Science and Technology, The Second Clinical Medical College of Jinan University). C57/BL6 mice (female, 42-day-old) were provided by Hufukang (Beijing, China). LLC cells (1 × 10^6^) were initially administered into the mammary fat pads of the mice. After tumor growth initiation (tumor size > 50 mm^3^), the mice were intratumorally injected with chemically modified miR101 mimic or control miRNA (20 nM in 50 µl PBS) (Ribobio Co., Guangzhou, China) once every other day for 18 d. Tumor size was determined through Vernier caliper weekly, and volume was calculated according to (length × width^2^)/2. Mice were euthanized, and tumors were harvested for determining protein levels and immunochemistry staining. The serum was collected for ELISA analysis of IL1A and IL6.

### Statistical analysis

Data were analyzed through GraphPad Prism V.7.01 (La Jolla, California, USA). IBM SPSS V.24.0 was applied to receiver operating characteristic (ROC) calculations, logistic regression, and cross-validation. *P* < 0.05 indicated statistical significance.

## Supplementary information

Figure S1

Figure S2

Figure S3

Figure S4

Supplement figure legends
