# Supplementary figures and images for: Hypoxic stress suppresses lung tumor-secreted exosomal miR101 to activate macrophages and induce inflammation
Source: Cell Death Dis. 2021 Aug 6;12(8):776. doi: 10.1038/s41419-021-04030-x (PMC8346509; doi:10.1038/s41419-021-04030-x)

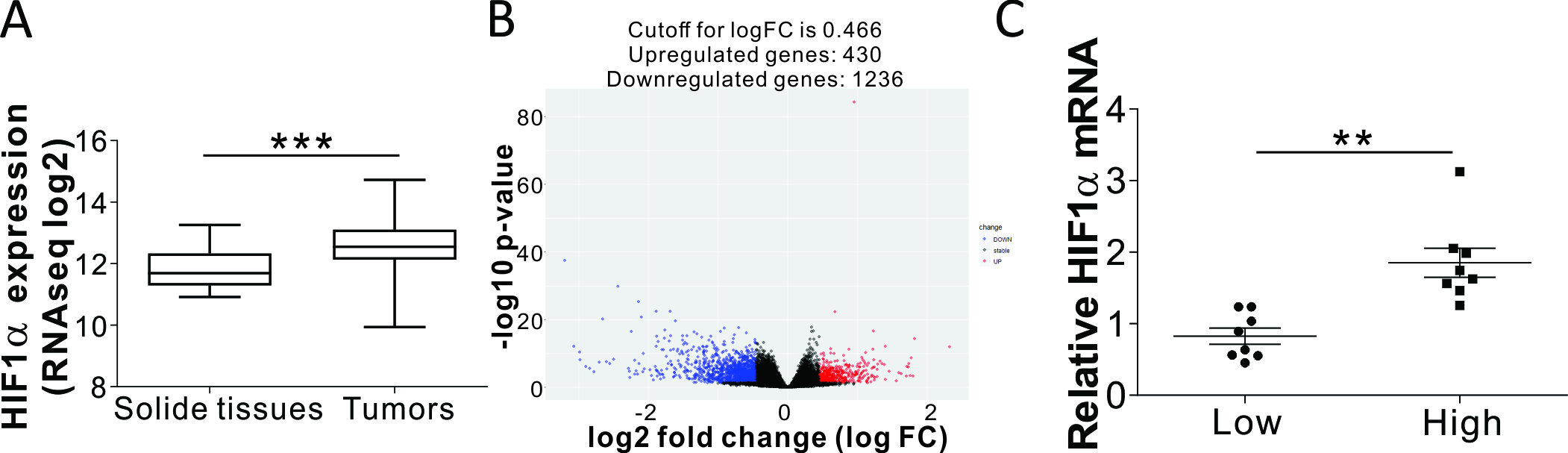

Supplement: Supplementary file 1 — Figure S1 [file 41419_2021_4030_MOESM1_ESM.tif]

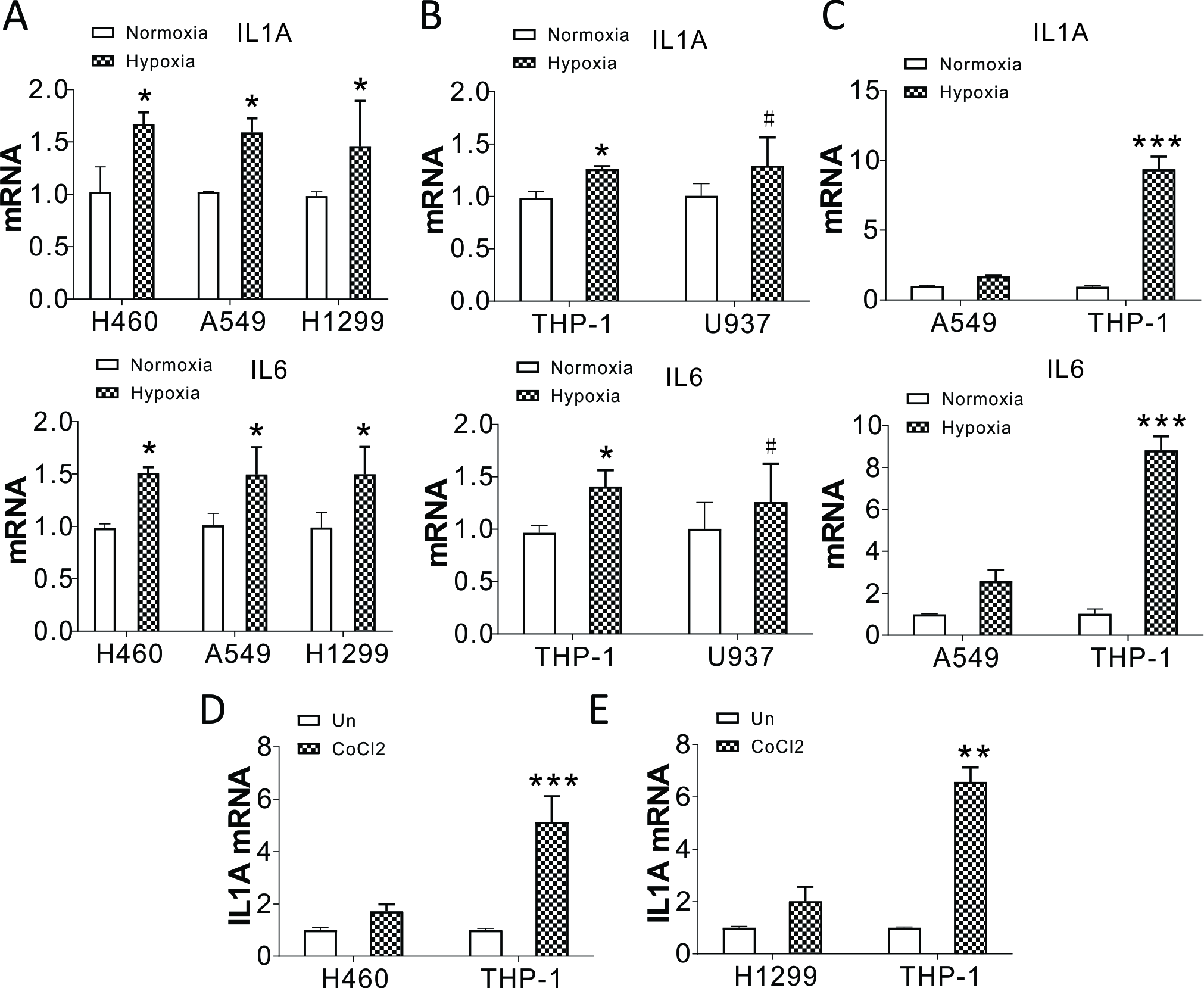

Supplement: Supplementary file 2 — Figure S2 [file 41419_2021_4030_MOESM2_ESM.tif]

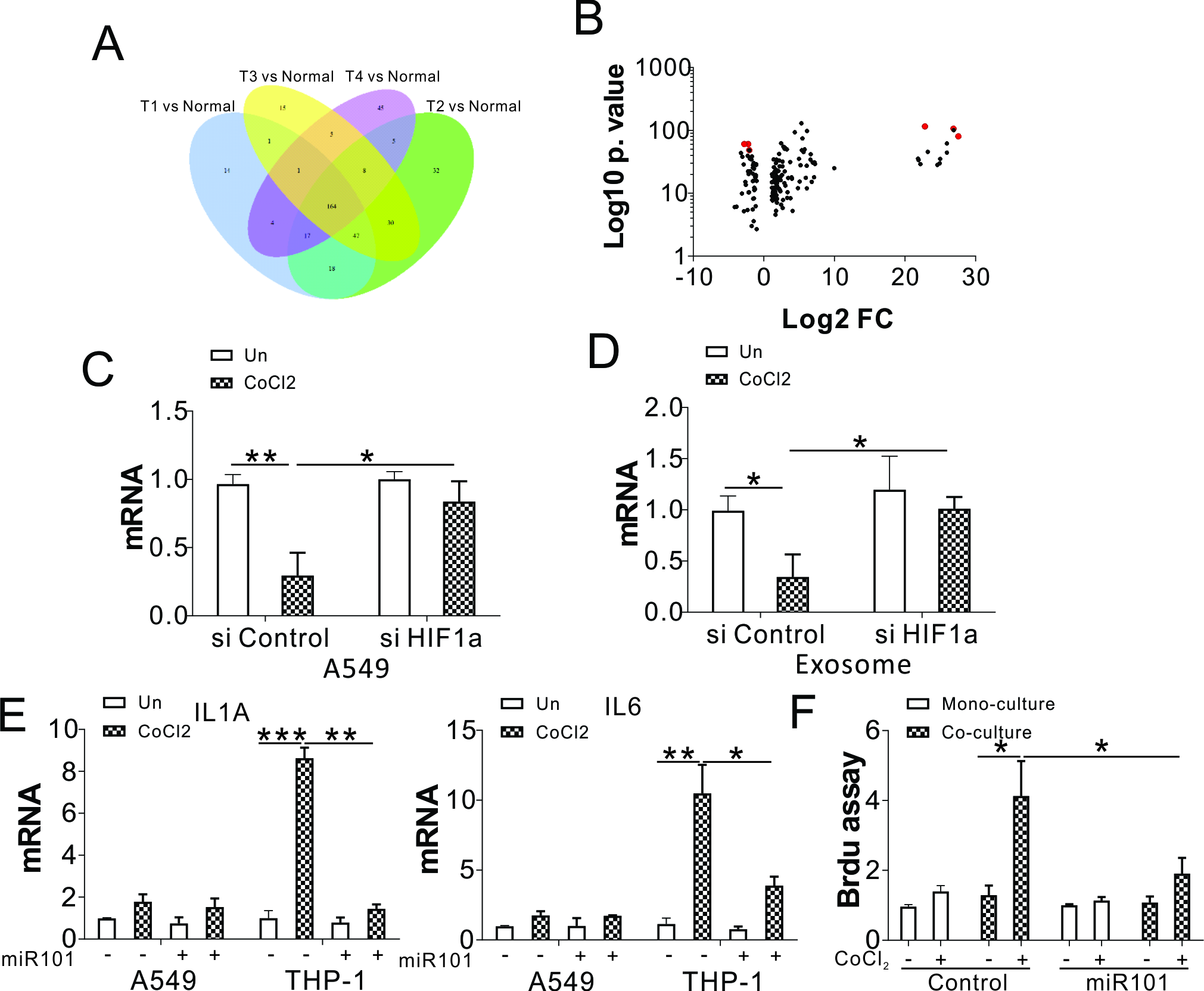

Supplement: Supplementary file 3 — Figure S3 [file 41419_2021_4030_MOESM3_ESM.tif]

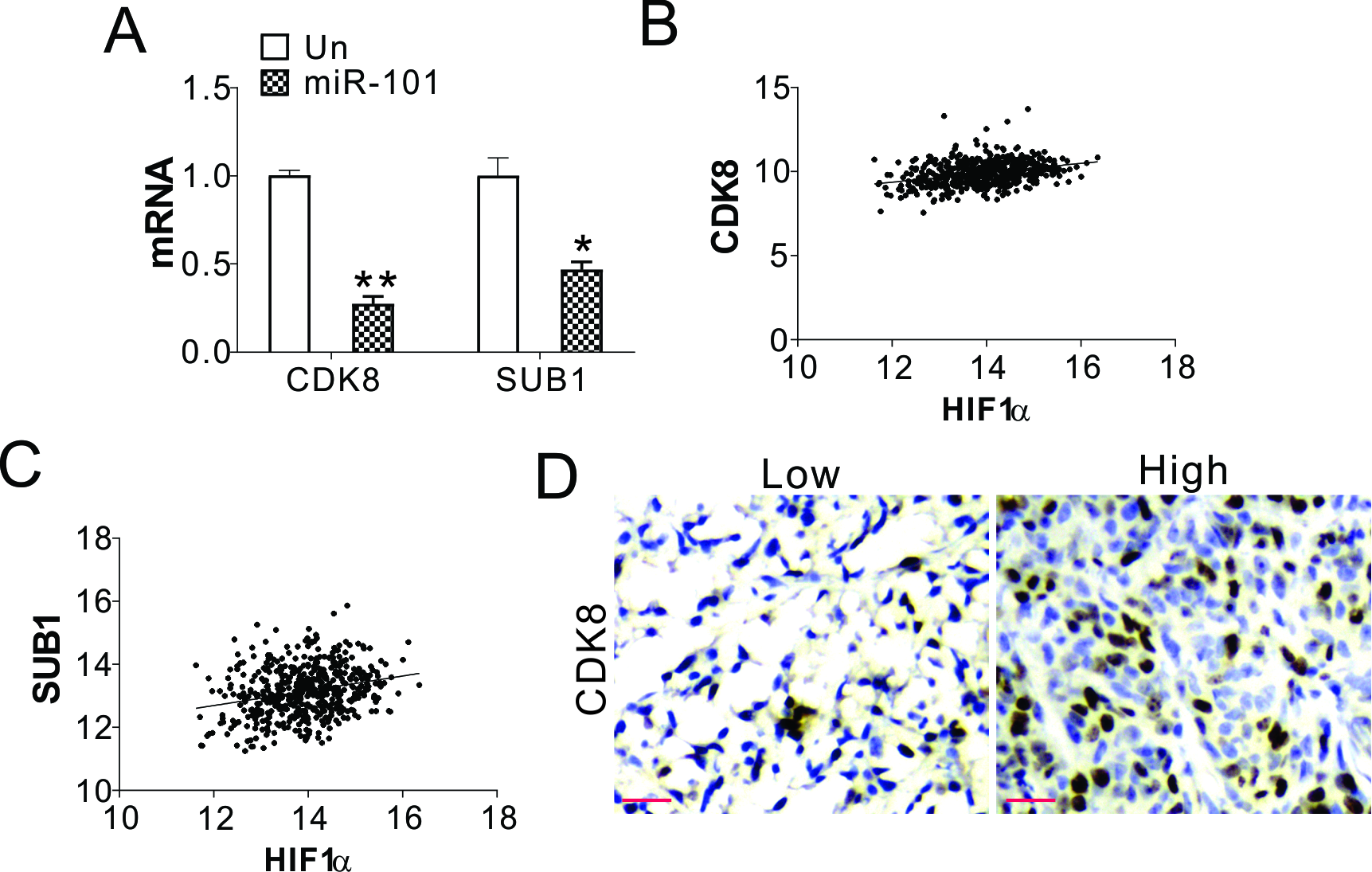

Supplement: Supplementary file 4 — Figure S4 [file 41419_2021_4030_MOESM4_ESM.tif]
