# Supplementary material for: Hypoxic stress suppresses lung tumor-secreted exosomal miR101 to activate macrophages and induce inflammation
Source: Cell Death Dis. 2021 Aug 6;12(8):776. doi: 10.1038/s41419-021-04030-x (PMC8346509; doi:10.1038/s41419-021-04030-x)
Supplement: Supplementary file 5 — Supplement figure legends [file 41419_2021_4030_MOESM5_ESM.docx]

**Fig. S1. The different expression of HIF1α in lung cancer.** **A.** The different expression of HIF1α in normal lung tissue and lung tumors based on the TCGA-LUSC RNA-seq database. **B.** The differentially expressed genes in HIF1α low and high expressed lung tumors. **C.** The mRNA level of HIF1α in 16 clinical samples from lung cancer patients. **, p<0.01; ***, p<0.001.

**Fig. S2. The expression of IL1A and IL6 under hypoxic stress.** **A.** The expression of IL1A and IL6 in H460, A549, and H1299 cultured in normaxia in hypoxia environment for 24 h. **B.** The expression of IL1A and IL6 in THP-1 and U937 cells cultured in normaxia in hypoxia environment for 24 h. **C.** The expression of IL1A and IL6 in A549 and THP-1 cells in the trans-well co-culture system in A549 cells cultured in normaxia in hypoxia environment for 24 h. **D.** IL1A expression in H460 and THP-1 cells in the trans-well co-culture system with or without CoCl_2_ treatment in H460 cells. **E.** IL1A expression in H1299 and THP-1 cells in the trans-well co-culture system with or without CoCl_2_ treatment in H1299 cells. #, p>0.05; *, p<0.05; **, p<0.01; ***, p<0.001.

**Fig. S3. The regulation of miR101 in lung cancer patients, cells, and exosomes.** **A.** The intersection of differentially expression miRNAs in different stages of lung cancer patients. Data was from TCGA-LUSC database. T1, stage 1; T2, stage 2; T3, stage 3; T4, stage 4. **B.** The differential expression miRNAs. **C.** The expression of miR101 in A549 cells transfected with siHIF1α with or without CoCl_2_ treatment. **D.** The expression of miR101 in exosome of A549 cells transfected with siHIF1α with or without CoCl_2_ treatment. **E, F.** A549 cells were treated with CoCl_2_ for 1 d, and co-cultured with THP-1 cells. The co-cultured cells were transfected with miR101 mimic. (E) The expression of IL1A and IL6 in A549 and THP-1 cells was analyzed by RT-PCR. (F) The growth of A549 was analyzed by BrdU assay. *, p<0.05; **, p<0.01; ***, p<0.001.

**Fig. S4. The correlation of CDK8 and SUB1 expression with HIF1α in TCGA database.** **A.** The mRNA level of CDK8 and SUB1 in A549 cells transfected with miR101 mimic. **B.** The correlation of CDK8 expression with HIF1α (p<0.001, R^2^=0.1097). **C.** The correlation of SUB1 expression with HIF1α (p<0.001, R^2^=0.0659). **D.** The immunochemistry staining of CDK8 in the lung tissues from HIF1α low and high expressed patients. The representative pictures were shown. Scale bar, 20 μm *, p<0.05; **, p<0.01.
